# Supplementary material for: Assessing Health and Economic Benefits of Omega-3 Fatty Acid Supplementation on Cardiovascular Disease in the Republic of Korea
Source: Healthcare (Basel). 2023 Aug 21;11(16):2365. doi: 10.3390/healthcare11162365 (PMC10454021; doi:10.3390/healthcare11162365)
Supplement: Supplementary file 1 [file healthcare-11-02365-s001.zip › Supplementary Figure S1.pdf]

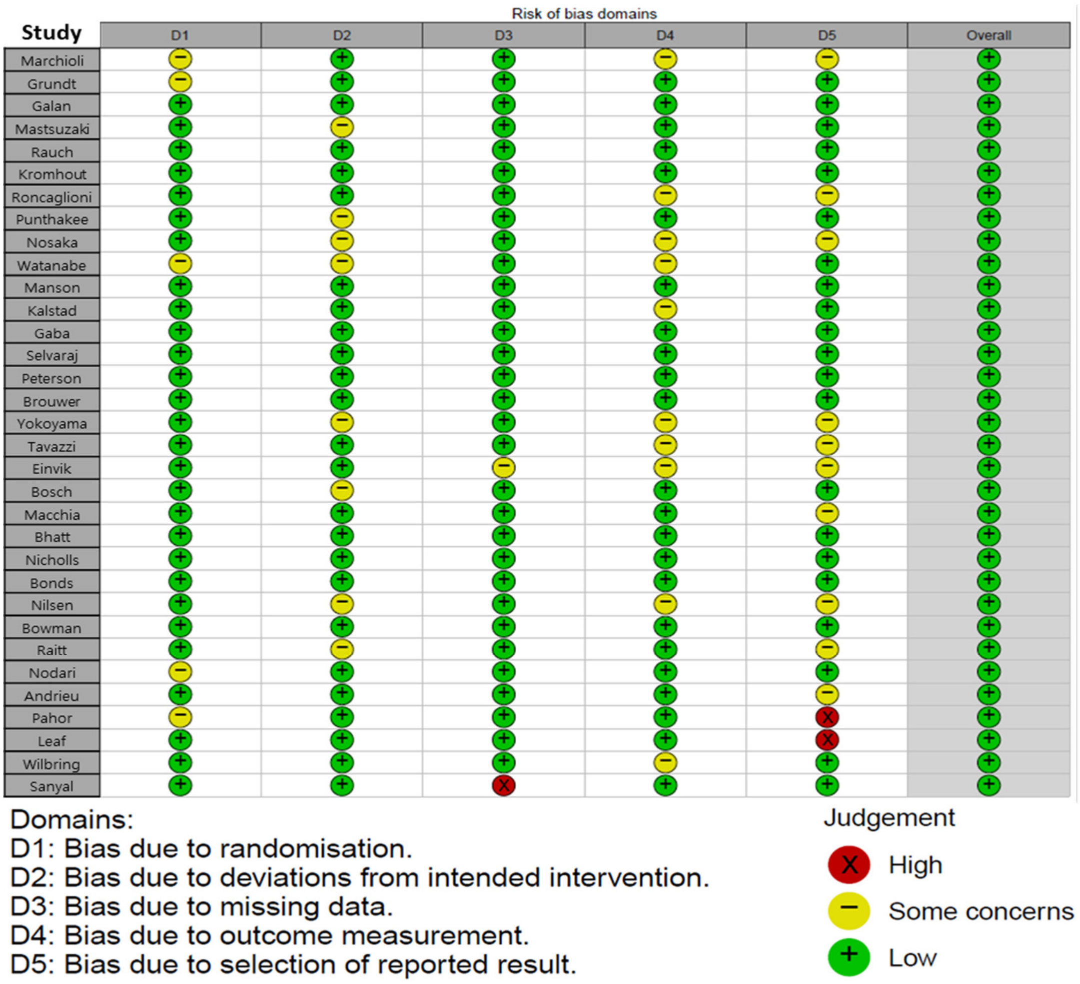

Figure S1 : Traffic light plot of risk of bias assessment of included studies using RoB 2.0 criteria, and overall risk of bias.
