# Supplementary material for: Assessing Health and Economic Benefits of Omega-3 Fatty Acid Supplementation on Cardiovascular Disease in the Republic of Korea
Source: Healthcare (Basel). 2023 Aug 21;11(16):2365. doi: 10.3390/healthcare11162365 (PMC10454021; doi:10.3390/healthcare11162365)
Supplement: Supplementary file 1 [file healthcare-11-02365-s001.zip › Supplementary Figure S2.pdf]

(a)

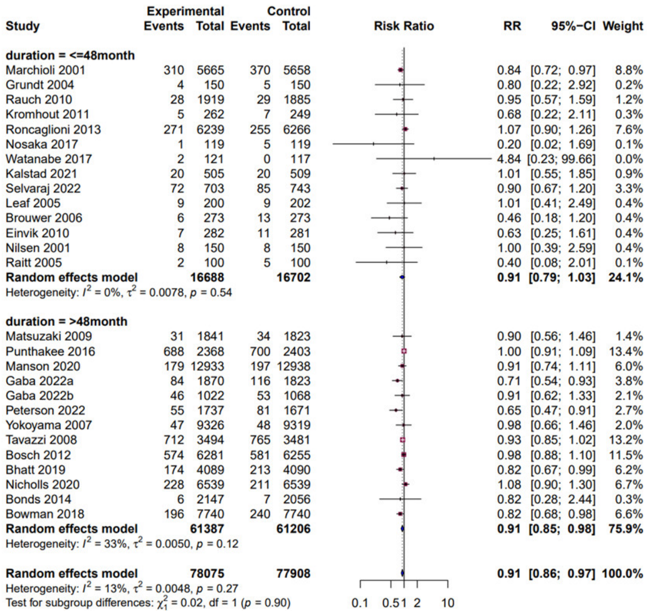

(b)

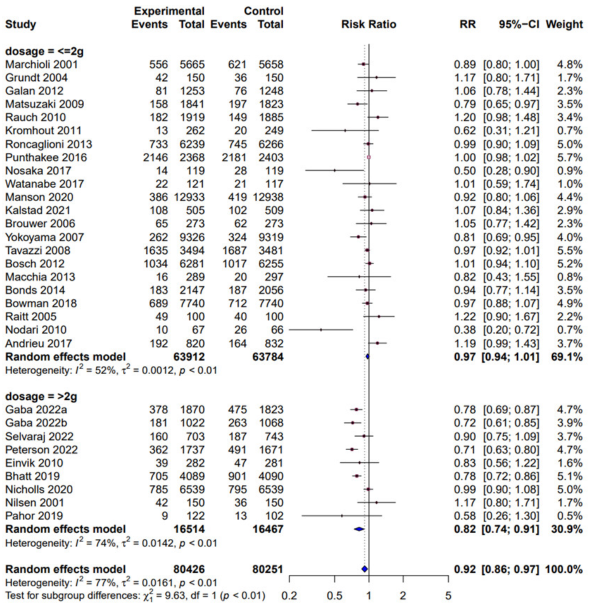

Figure S2 : Subgroup analysis for all cardiovascular diseases according to (a) duration (2 years or less vs. more than 2 years) and dosage (2g or less vs. more than 2g). Experimental: omega 3 intervention group, event: CVD incidence, CI: confidence interval; RR: risk ratio
