# Supplementary material for: Assessing Health and Economic Benefits of Omega-3 Fatty Acid Supplementation on Cardiovascular Disease in the Republic of Korea
Source: Healthcare (Basel). 2023 Aug 21;11(16):2365. doi: 10.3390/healthcare11162365 (PMC10454021; doi:10.3390/healthcare11162365)
Supplement: Supplementary file 1 [file healthcare-11-02365-s001.zip › Supplementary Table S1.pdf]

Table S1 : Searching queries in three database

Database	Search string
PubMed	(("fatty acids, omega 3"[MeSH Terms] OR ("fatty acids, omega 3"[MeSH Terms] OR ("fatty"[All Fields] AND "acids"[All Fields] AND "omega 3"[All Fields]) OR "omega-3 fatty acids"[All Fields] OR "omega 3"[All Fields]) OR "n3"[All Fields] OR ("PUFA"[All Fields] OR "polyunsaturated fatty acid"[All Fields]) OR ("eur policy anal"[Journal] OR "epa"[All Fields]) OR "DHA"[All Fields]) AND ("myocardial infarction"[MeSH Terms] OR ("stroke"[MeSH Terms] OR "stroke"[All Fields] OR "strokes"[All Fields] OR "stroke s"[All Fields]) OR ("coronaries"[All Fields] OR "heart"[MeSH Terms] OR "heart"[All Fields] OR "coronary"[All Fields]) OR ("myristica"[MeSH Terms] OR "myristica"[All Fields] OR "mace"[All Fields] OR "major adverse cardiovascular event"[All Fields])) AND ("randomized controlled trial"[Publication Type] OR "randomized controlled trial"[All Fields] OR ("double blind method"[MeSH Terms] OR ("double blind"[All Fields] AND "method"[All Fields]) OR "double blind method"[All Fields] OR ("double"[All Fields] AND "blind"[All Fields]) OR "double blind"[All Fields]) OR ("single blind method"[MeSH Terms] OR ("single blind"[All Fields] AND "method"[All Fields]) OR "single blind method"[All Fields] OR ("single"[All Fields] AND "blind"[All Fields]) OR "single blind"[All Fields]) OR ("multicenter"[All Fields] OR "multicentered"[All Fields] OR "multicenters"[All Fields]) OR "random allocation"[All Fields])) AND (2000:2022[pdat])
Embase	(('omega 3 fatty acid'/exp OR 'omega 3 fatty acid') OR 'omega 3' OR (pufa OR 'polyunsaturated fatty acid') OR epa OR dha) AND ('heart infarction'/exp OR stroke OR coronary OR (mace OR 'major adverse cardiovascular event')) AND ('randomized controlled trial'/exp OR 'double blind' OR 'single blind' OR multicenter OR 'random allocation') AND [01-01-2000]/sd NOT [11-10-2022]/sd
Cochrane Library	(MeSH descriptor: [Fatty Acids, Omega-3] explode all trees OR omega-3 OR n3 OR PUFA OR "polyunsaturated fatty acid" OR EPA OR DHA) AND (“myocardial infarction” OR MeSH descriptor: [Myocardial Infarction] explode all trees OR stroke OR coronary OR MACE OR "major adverse cardiovascular event") AND (“double blind” OR “randomized controlled trial” OR multicenter OR “single blind” OR “random allocation”)<br>Limits : with Cochrane Library publication date Between Jan 2000 and Oct 2022, trials
