# Supplementary material for: Assessing Health and Economic Benefits of Omega-3 Fatty Acid Supplementation on Cardiovascular Disease in the Republic of Korea
Source: Healthcare (Basel). 2023 Aug 21;11(16):2365. doi: 10.3390/healthcare11162365 (PMC10454021; doi:10.3390/healthcare11162365)
Supplement: Supplementary file 1 [file healthcare-11-02365-s001.zip › Supplementary Table S2.pdf]

Table S2: Estimation equation of costs in this study

Category	Source	Equation
Direct cost	Medical	$M=\sum_{j}\sum_{k}H_{jk}*N_{jk}$<br><br>*M*: direct medical costs, *H*= cost of nursing care per capita, *N* = target population, *j*= outpatient group, *k*= inpatient group
	Non-Medical	$NM=\sum_{j}T_{j}\times L_{j}+[(\sum_{k}A_{k}+\sum_{k}LC_{k})\times L_{k}]$<br><br>*NM* = direct non-medical cost, *T* = average transportation cost per time, *L* = number of treatment days, *A* = average nursing care cost per day, *LC* = average leisure cost per day, *j*= outpatient group, *k*= inpatient group
Indirect cost	Productivity Loss	$P=\sum_{j}\sum_{k}N_{j,k}\times E\times Y\times[\frac{L_{k}}{N_{k}}+(\frac{L_{j}}{N_{j}}\times\frac{1}{3})]$<br><br>*P* = cost of lost productivity, *N* = target population, *E* = employment rate, *Y* = average daily wage, *L* = number of days of treatment, *j*= outpatient group, *k*= inpatient group
	Loss due to premature death	$D=\sum_{j}\sum_{k}N_{j,k}\times E\times Y\times F\times R$<br><br>*D* = loss cost due to premature death, *N* = target population, *E* = employment rate, *Y* = average annual labor cost, *F* = duration of activity (assumed to be 5 years), *R* = death rate from CVD disease, j = outpatient group, k = inpatient group
Purchase expense		Daily omega-3 intake (g/day) × average selling price (won/g) × number of CVD population aged 50 or older (persons)
